# Comparison of endoscopic and pathological findings of the upper gastrointestinal tract in transplant candidate patients undergoing hemodialysis or peritoneal dialysis treatment: a review of literature

**DOI:** 10.1186/s12882-020-02108-w

**Published:** 2020-10-22

**Authors:** Mehmet Usta, Alparslan Ersoy, Yavuz Ayar, Gökhan Ocakoğlu, Bilgehan Yuzbasioglu, Emrullah Düzgün Erdem, Omer Erdogan

**Affiliations:** 1 Bursa City Hospital, Department of Nephrology, Nephrology Clinic, Dogankoy mevki, Nilufer, Bursa, Turkey; 2 Division of Nephrology, Department of Internal Medicine, Bursa Uludag University Faculty of Medicine, Gorukle, Nilufer, Bursa, Turkey; 3 Faculty of Medicine, Department of Bioistatistics, Uludag University, Gorukle, Nilufer, Bursa, Turkey; 4 Bursa State Hospital, Department of Gastroenterology, Gastroenterology Clinic, Osmangazi, Bursa, Turkey; 5 Department of Pathology, Bursa City Hospital, Dogankoy mevki, Nilufer, Bursa, Turkey

**Keywords:** Hemodialysis, Peritoneal dialysis, Transplant waiting list, Endoscopy, Gastritis, Helicobacter pylori

## Abstract

**Background:**

Dyspepsia is a common disorder in kidney transplant recipients, and the risk of post-transplant complications is increased in candidates with upper gastrointestinal disease. We evaluated gastrointestinal lesions of kidney transplant candidates on dialysis.

**Methods:**

In this study, endoscopic and pathological findings in hemodialysis (HD) and peritoneal dialysis (PD) patients with gastrointestinal symptoms on the waiting list were compared.

**Results:**

The most common non-ulcerous lesions in the endoscopic examination were gastritis (62.3%), erosive gastritis (38.7%), duodenal erosion or duodenitis (18.9%) and esophagitis (13.2%). The ulcerous lesion was present in only 3 patients. Gastroesophageal reflux disease, ulcerated lesion and non-ulcerated lesion rates were similar in both dialysis groups. Histopathological examination revealed *Helicobacter pylori* (HP) positivity in 28.3% of patients. HP positivity rate was significantly higher in PD patients than in HD patients (38.7% vs. 13.6%, *p* = 0.046). Chronic gastritis (75.5%) was the most common pathological finding. HP positivity rate was 37.5% in patients with chronic gastritis, but HP was negative in patients without chronic gastritis. In multivariate analysis, male gender, urea and albumin levels were associated with the presence of pathological chronic gastritis. The presence of gastritis, total cholesterol and ferritin levels were found significant for HP positivity. A total cholesterol > 243 mg/dL was significantly related to an increased risk of the presence of HP positivity.

**Conclusions:**

Gastrointestinal lesions and HP infection are common in dialysis patients. Dialysis modality may affect the frequency of some lesions. It may be useful to have an endoscopic examination before entering the transplant waiting list for all candidates.

## Background

Dyspeptic complaints such as vomiting, anorexia, abdominal pain, souring and belching are common in patients with chronic kidney disease [1, 2]. Uremic patients have a high risk of gastroduodenal discomfort due to high urea levels, increased gastrin level, gastric acid hypo- and hypersecretion, decreased gastrointestinal motility, amyloid deposition, and *Helicobacter pylori* (HP) infection [2–4]. However, the deterioration in psychological general well-being may be associated with a high prevalence of gastrointestinal symptoms in patients with chronic kidney disease (CKD), regardless of presence of dialysis or diabetes mellitus or not, and type of dialysis [1].

HP infection, which is common all over the world and in our country [5, 6], can cause gastritis, peptic (gastric or duodenal) ulcer, gastric cancer and gastric lymphoma (primary gastric B cell lymphoma, MALT lymphoma) [7, 8]. Although some studies report the prevalence of HP infection in dialysis patients to be lower than the normal population [9, 10], it ranges from 15 to 74.4% in normal population and 20 to 64% in CKD patients [11–13]. Recently, many studies have also shown that HP infection plays an important role in the development of gastrointestinal complications such as heartburn, peptic ulcer disease, gastric erosion in hemodialysis (HD) or continuous ambulatory peritoneal dialysis (PD) patients [9, 14, 15]. The risk of malignancy in dialysis patients is also increased. Endoscopic examination of dyspeptic symptoms is the most appropriate diagnostic approach. Candidate patients on the kidney transplant waiting list are carefully evaluated for absolute and relative contraindications before transplantation. The transplant recipients are susceptible to a variety of gastrointestinal complications such as infections, ulcer disease and malignancies [16]. In addition, immunosuppressive drugs, especially corticosteroids, used after kidney transplantation have adverse effects on the gastrointestinal tract (peptic ulcer bleeding, perforation, gastritis and esophagitis). Therefore, it is recommended that transplant candidates with gastrointestinal complaints or suspected malignancy should be undergo endoscopy prior to transplantation and treated if necessary. This study aimed to evaluate the results of upper gastrointestinal endoscopy in dialysis patients who are candidates for kidney transplantation.

## Methods

We evaluated adult 129 dialysis patients (45 PD, 84 HD) over 18 years of age who were candidates for kidney transplantation. Among all patients, 53 patients (34 males, 19 females) who underwent upper gastrointestinal endoscopy for gastrointestinal symptoms were included in the study. Ethical approval was obtained, and all participants provided written informed consent. Patients were divided into two groups: PD (*n* = 31, 20 males, 11 females) and HD (*n* = 22, 14 males, 8 females) groups. Anti-HCV and anti-HIV were negative in all patients. In the HD group, 1 patient had hepatitis B positivity. The clinical characteristics, esophagogastroduodenoscopy findings and biopsy (histological evaluation and identification of HP-staining) results were obtained from the medical records, retrospectively.

### Statistical analysis

A Shapiro–Wilk test was used to assess whether the variables followed normal distribution. Categorical variables were given as number and percentage, and continuous variables as mean ± standard deviation (SD) or median (min:max). According to the normality test results, Mann Whitney U test or independent samples t-tests were used to compare the groups. Categorical variables were compared by Chi square test and Fisher’s exact test. To determine the independent risk factors affecting the presence of endoscopic gastritis and duedonitis and pathological chronic gastritis and HP, binary logistic regression analysis was performed. The covariates that had a *P* value < 0.2 in the univariate analysis were included in the multivariable logistic regression analysis, and forward selection and backward elimination method was used in finding the best parsimonious final model. In order to estimate the sensitivity and specificity of hemoglobin, urea and total cholesterol for predicting the presence of duodenitis, chronic gastritis and HP positivity receiver operator characteristic (ROC) curve analysis was performed. The data was analyzed using the SPSS Software (IBM Corp. Released 2015. IBM SPSS Statistics for Windows, Version 23.0. Armonk, NY: IBM Corp). A *p* value of < 0.05 was considered statistically significant.

### Conflict of interest

There is no conflict of interest in our study. The study was retrospective and no funding was received.

## Results

The mean age in the HD group was higher than in the PD group [65.4 ± 11.5 (30–79) vs. 56.2 ± 13.2 (29–86) years, *p* = 0.005]. The mean dialysis durations were similar in the HD [46.0 ± 39.6 (6.96–171.2) months] and PD [42.0 ± 45.9 (1.25–138.6) months] groups (*p* = 0.295). Diabetes melllitus ratio (38.7% vs. 27.3%), gender and primary disease distributions were not different in the PD and HD groups, respectively. The hypertension ratio in PD group was higher than HD group (67.7% vs. 4.5%, *p* < 0.001). When compared with PD group, Kt/V value (1.76 ± 0.35 vs. 2.21 ± 0.35, p < 0.001), serum calcium levels (9.10 ± 0.63 vs. 9.66 ± 0.87 mg/dL, *p* = 0.017) and total iron binding capacity was lower (95.7 ± 53.7 vs. 201.7 ± 47.7 mcg/dL, p < 0.001), and transferrin saturation (TSAT: 237 ± 317 vs. 39.8 ± 15.6%, *p* = 0.001) and ferritin (758 ± 701 vs. 235 ± 191 mcg/L, *p* = 0.011) levels were higher in HD group, respectively. There was no significant difference between serum creatinine, uric acid, glucose, HbA1c, lipid profile, electrolytes, total protein, albumin, liver function tests, lypase, iron, phosphorus, calcium x phosphorus product (CaxP), alkaline phosphatase, parathormone, hemoglobin and C-reactive protein (CRP) levels in both dialysis groups (*p* > 0.05).

In endoscopic examination; only 3 patients had an ulcerous lesion, while all patients had non-ulcerous lesions. There was no difference between gastroesophageal reflux disease (GERD), ulcerated lesion and non-ulcerated lesion rates in both dialysis groups (Table 1). According to histopathological findings, the frequency of HP positivity was significantly higher in the PD group (*p* = 0.046). However, the frequency of other pathological findings was similar in both dialysis groups. No patient had dysplasia (Table 2).

**Table 1:** Comparison of endoscopic findings in groups

Variables, n(%)	Total (***n*** = 53)	PD (***n*** = 31)	HD (***n*** = 22)	***p***-value
Gastroesophageal reflux disease	22 (41.5%)	14 (45.2%)	8 (36.4%)	0.522^a^
Non-ulcerous lesion				
Gastritis	33 (62.3%)	19 (61.3%)	14 (63.6%)	0.862^a^
Erosive gastritis	19 (35.8%)	12 (38.7%)	7 (31.8%)	0.606^a^
Esophagitis	7 (13.2%)	6 (19.4%)	1 (4.5%)	0.218^b^
Duodenal erosion/duodenitis	10 (18.9%)	6 (19.4%)	4 (18.2%)	> 0.99^b^
Polyp	2 (3.8%)	1 (3.2%)	1 (4.5%)	> 0.99^b^
Esophageal varicosis	1 (1.9%)	0	1 (4.5%)	0.415^b^
Ulcerous lesion	3 (5.7%)	2 (6.5%)	1 (4.5%)	> 0.99^b^
Gastric ulcer	1 (1.9%)	1 (3.2%)	0	> 0.99^b^
Duodenal ulcer	2 (3.8%)	1 (3.2%)	1 (4.5%)	> 0.99^b^
Pyloric diverticulum	1 (1.9%)	0	1 (4.5%)	0.415^b^

^a^: Chi-square test, ^b^: Fisher’s exact test

**Table 2:** Comparison of pathological findings in groups

Variables, n(%)	Total (***n*** = 53)	PD (***n*** = 31)	HD (***n*** = 22)	***p***-value
Chronic gastritis	40 (75.5%)	24 (77.4%)	16 (72.7%)	0.696^a^
Non-spesific gastritis	13 (24.5%)	7 (22.6%)	6 (27.3%)	0.696^a^
Intestinal metaplasia	9 (17%)	5 (16.1%)	4 (18.2%)	> 0.99^b^
Atrophy	8 (15.1%)	3 (9.7%)	5 (22.7%)	0.253^b^
Helicobacter pylori	15 (28.3%)	12 (38.7%)	3 (13.6%)	**0.046**^**a**^
Serrated adenoma	1 (1.9%)	0	1 (4.5%)	0.415^b^
Hyperplastic polyp	2 (3.8%)	1 (3.2%)	1 (4.5%)	> 0.99^b^
Dysplasia	0	0	0	–

^a^:Chi-square test, ^b^: Fisher’s exact test

The characteristics of dialysis patient with (*n* = 40) and without (*n* = 13) chronic gastritis were compared. The HP positivity rate in the group with chronic gastritis was 37.5% (*n* = 15). HP was negative in the group without chronic gastritis (*p* = 0.011). In the group with chronic gastritis, median sodium [140(129:145) vs. 138(133:142) mmol/L, *p* = 0.033) and LDL cholesterol [146(77:217) vs. 118(54:180) mg/dL, *p* = 0.043)] and mean total protein (6.76 ± 0.58 vs. 6.24 ± 0.72 g/dL, *p* = 0.012) and albumin (4.07 ± 0.39 vs 3.77 ± 0.43 g/dL, *p* = 0.027) levels were higher. Mean urea (98.4 ± 23 vs. 125.2 ± 38.7 mg/dL, *p* = 0.032) and median ferritin [191.5(10.4:2000) vs. 358(62.4:1799) mcg/L, *p* = 0.043)] levels were lower in the group with chronic gastritis.

HP infection was detected in 28.3% of dialysis patients. The characteristics of dialysis patient with (*n* = 15) and without (*n* = 38) HP were compared. In endoscopic examination, gastritis rate in the HP positive group (86.7% vs. 52.6%, *p* = 0.021) was higher than negative group and erosive gastritis rate (44.7% vs. 13.3%, *p* = 0.032) was higher in HP negative group than positive group. In histopathological examination, while chronic gastritis was observed in all HP positive group (100%), the rate of chronic gastritis in the HP negative group was 65.8% (*n* = 25, *p* = 0.011). There was no intestinal metaplasia in the HP positive group. This rate was 23.7% (*n* = 9) in the HP negative group (*p* = 0.047). The mean total cholesterol level in the HP positive group was higher than the HP negative group (229.6 ± 39.9 vs. 202.6 ± 37.1 mg/dL, *p* = 0.023). Median dialysis durations of HP positive [22.0(1.25:171.2) month] and negative [30.3(1.31:171.2) month] groups were comparable (*p* = 0.984).

### Risk analysis

The independent risk factors affecting the development of endoscopic gastritis and duedonitis and pathological chronic gastritis and HP were determined with multivariable logistic regression analysis. The logistic regression models obtained in the final step of the analysis were found significant and the data set was compatible with the models. Regression analysis models for each dependent variable included the following variables: pathological chronic gastritis, HP positivity, hypertension, triglyceride, LDL cholesterol and CRP for the presence of endoscopic gastritis (Model χ2 = 9.17; *p* = 0.010, R2 = 21.6%, Hosmer and Lemeshow Test: *p* = 0.183); age, dialysis duration, GERD, Kt/V, sodium, chloride, total cholesterol, hemoglobin, calcium, phosphorus, CaxP and lipase for the presence of endoscopic duedonitis (Model χ2 = 30.55; *p* < 0.001, R2 = 58.4%, Hosmer and Lemeshow Test: *p* = 0.492); gender, endoscopic ulcerous lesion and gastritis, Kt/V, urea, sodium, chloride, total protein, albumin, LDL cholesterol, TSAT, ferritin, phosphorus and CaxP for the presence of pathological chronic gastritis (Model χ2 = 18.45; *p* < 0.001, R2 = 43.8%, Hosmer and Lemeshow Test: *p* = 0.623) and gastritis, dialysis type, erosive gastritis, urea, HbA1c, total cholesterol, LDL cholesterol, ferritin, calcium and gamma glutamyltransferase (GGT) for the presence of HP positivity (Model χ2 = 26.44; p < 0.001, R2 = 56.4%, Hosmer and Lemeshow Test: *p* = 0.872). In the multivariate analysis, only HP positivity (*p* = 0.040) influenced the presence of endoscopic gastritis. Hemoglobin (*p* = 0.027) and chloride levels (*p* = 0.013) influenced the presence of endoscopic duedonitis. Men gender (*p* = 0.030), urea (*p* = 0.014) and albumin levels (*p* = 0.048) influenced the presence of pathological chronic gastritis. The presence of gastritis (*p* = 0.040), total cholesterol (*p* = 0.006) and ferritin levels (*p* = 0.048) influenced HP positivity. Variables in the models formed in the final step of multivariable logistic regression analysis were showed in Table 3.

**Table 3:** Multivariable logistic regression analysis of independent risk factors affecting the development of endoscopic gastritis and duedonitis and pathological chronic gastritis and HP positivity

	Wald	***p***-value	Odds ratio (OR)	95% CI	
				Lower	Upper
**Gastritis**					
HP (presence)	4.22	**0.040**	5.71	1.08	30.05
CRP	2.17	0.140	0.87	0.72	1.05
**Duodenitis**					
GERD (presence)	2.84	0.092	4.02	0.80	20.30
Chloride	6.22	**0.013**	0.90	0.83	0.98
Hemoglobin	4.87	**0.027**	2.21	1.09	4.45
**Chronic gastritis**					
Gender (male)	4.69	**0.030**	9.90	1.24	78.83
Urea	6.01	**0.014**	0.95	0.91	0.99
Albumin	3.90	**0.048**	8.83	1.02	76.79
**HP positivity**					
Gastritis (presence)	4.20	**0.040**	9.50	1.10	81.90
Total cholesterol	7.59	**0.006**	1.04	1.01	1.06
GGT	3.08	0.079	0.91	0.83	1.01
Ferritin	3.93	**0.048**	0.97	0.97	0.99

*HP* Helicobacter pylori, *CI* confidence interval, *CRP* C-reactive protein, *GERD* gastroesophageal reflux disease, *GGT* gamma glutamyltransferase (GGT)

**Fig. 1:**
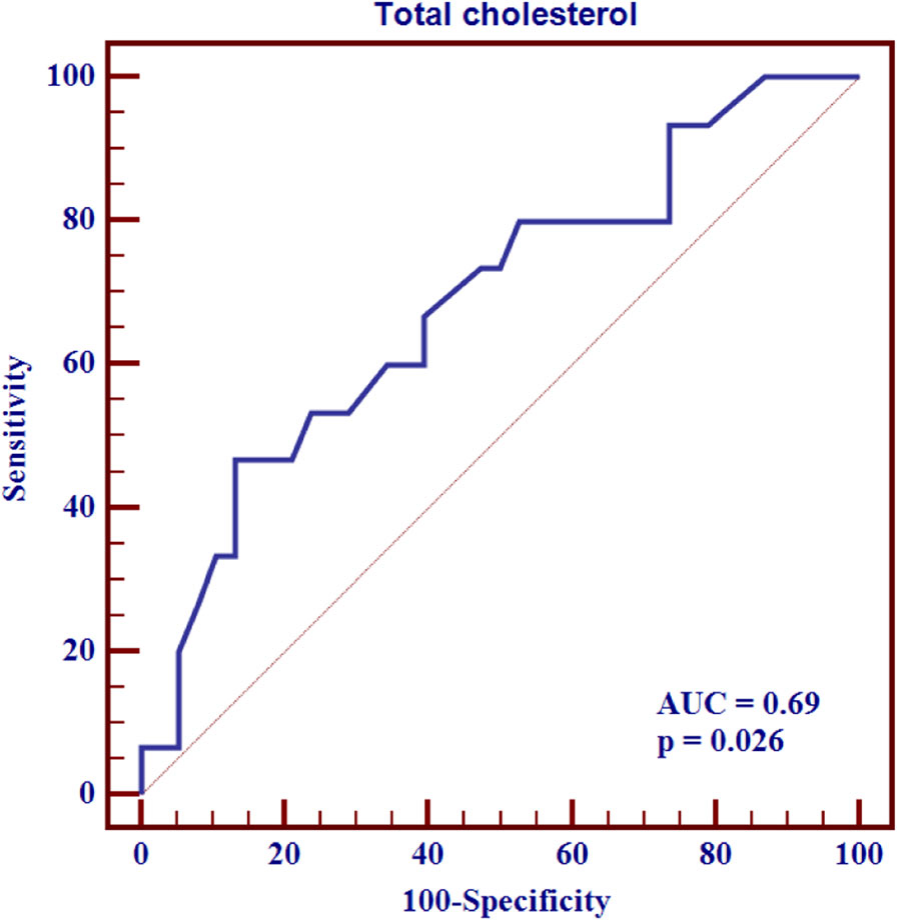
The realtioship between total cholesterol and *Helicobacter pylori* positivity

In ROC curve analysis, the cut-off point for hemoglobin was determined as > 10.7 g/dL for the sensitivity and specificity of hemoglobin for predicting the presence of duodenitis. The area under the curve for hemoglobin was 0.68 (sensitivity 100%, specificity 39.53%, *p* = 0.022). The cut-off point for urea was determined as ≤112 mg/dL for the sensitivity and specificity of urea for predicting the presence of chronic gastritis. The area under the curve for urea was 0.72 (sensitivity 80%, specificity 61.54%, *p* = 0.017). The cut-off point for total cholesterol was determined as > 243 mg/dL for the sensitivity and specificity of total cholesterol for predicting the presence of HP positivity (Fig. 1). The area under the curve for total cholesterol was 0.69 (sensitivity 46.67%, specificity 86.84%, *p* = 0.026).

## Discussion

The incidence of gastrointestinal disorders is high in patients with CKD due to increased risk of gastric mucosal damage associated with high ammonia levels, systemic and/or local chronic circulatory failure and hypergastrinemia [17]. In 5161 dialysis patients (3804 HD and 1507 PD), the most prevalent symptoms were constipation, indigestion, abdominal pain and reflux [18]. However, there is no good correlation between the symptoms of the patients and endoscopic gastroduodenal lesions [19, 20]. In one study, 73.8% of the patients were asymptomatic, whereas 35.9% of the patients had normal endoscopy [20]. In contrast, some studies report lower rates of normal mucosal findings on endoscopy in HD patients, ranging from 9.3 to 17.5% [21, 22]. Asymptomatic gastroduodenal lesions such as erosive gastroduodenitis and peptic ulcer may be seen in 46% of end-stage renal disease (ESRD) patients [23]. There may be a positive correlation between upper gastrointestinal lesions and CKD severity. Upper gastrointestinal lesions are seen in 80% of patients with advanced CKD and dialysis [24].

Despite similarities, the definition and frequency of esophagagastroduodenal lesions in endocopic examination varies in most studies. Abnormal endoscopic findings of patients on maintenance HD were erosive esophagitis (30.2%), gastroesophageal reflux disease (GERD, 10%), esophagitis (5.8%), enanthematous pangastritis (57.3%), diffuse antral erythema (27.8%), gastric erosion (58%), gastric antral erosion (22.8%), gastric intestinal metaplasia (8.33%), gastritis and duodenitis (42%), duodenal erosion (18–32%), nodular duodenum (2%), peptic ulcer (7.3%), gastric ulcer (7–14%), duodenal ulcer (7.3–18.4%), angiodysplasia (4.4%) and inflammatory gastric polyps (1.5%) in different studies [19, 20, 25–27]. In a large dialysis cohort, endoscopic examination revealed 77.8% gastritis, 11.4% gastric ulcer, 6.4% duodenal ulcer and 1.7% gastric cancer. On the other hand, 92.6% gastritis, 4.1% gastric ulcer and 3.3% duodenal ulcer were detected in the annual health checks of patients with normal renal function [9]. In endoscopic examination, hiatus hernia (29.3% vs. 14%) was more frequent and duodenal ulcer (3.26% vs. 16%) was lower in dialysis patients compared with non-uremic dyspeptic patients [28]. In our cohort, the most common non-ulcerous lesions in the endoscopic examination of dialysis patients with dyspeptic symptoms were gastritis (62.3%), erosive gastritis (38.7%), duodenal erosion/duodenitis (18.9%) and esophagitis (13.2%). When the frequency of gastrointestinal disorders was compared in 8955 non-uremic patients, 1791 PD and 8955 HD patients, GERD, abdominal hernia, intestinal obstruction or adhesions in PD group and peptic ulcer disease, lower gastrointestinal diverticulum and hemorrhage in HD group was significantly higher than in other groups [29]. Among 422 patients with ESRD on maintenance dialysis 322 were endoscopied, and patients who had erosive pre-pyloric changes of grade 2 and 3 were older, receiving dialysis longer, and more likely to be on HD rather than PD when compared with patients without erosive pre-pyloric changes [30]. More exposure to inflammatory conditions and oxidative stress in HD can damage or aggravate the stomach or small intestine mucosa. The other potential reasons of peptic ulcer in HD patients may be anticoagulant use, intradialytic hypotension and hemodynamic changes [31]. Ulcerous lesions were found in our 3 patients (5.7%). One patient with hepatitis B had only esophageal varicosis. However, the difference between the frequency of endoscopic lesions in PD and HD patients was not significant.

GERD is a common upper gastrointestinal disorder in patients with CKD (77.5%) and kidney transplant (75%) patients than normal population (38.6%) with the same upper gastrointestinal symptoms, while HP infection was lower (40, 36.1 and 75%, respectively). Although there was no significant difference between the prevalences of GERD (33.3 and 39.5%), erosive reflux esophagitis (16.7 and 23.7%) and non-erosive reflux disease (16.7 and 13.2%) in PD and HD, respectively, their prevalences were higher than those of the general population [32–34]. CKD, PD, gastrointestinal amyloidosis, immunosuppression and absence of HP infection seem to be risk factors for the development of GERD in ESRD patients [32, 35]. Some studies found to be similar the prevalences of GERD in ESRD patients and non-CKD controls with the same upper gastrointestinal symptoms [34, 35]. The frequency of GERD in our patients was 41.5%. GERD rate was higher in PD patients than in HD patients (45.2% vs. 36.4%), but this difference did not reach statistical significance. PD patients had more severe reflux and eating dysfunction than HD and pre-dialytic patients [1, 36]. Higher prevalence of GERD has been shown in PD patients than in HD patients (44.6% vs. 19.8%). This may be due to increased intraperitoneal pressure by PD [37].

In various studies, erosive gastritis, atrophic gastritis, and intestinal metaplasia was found in normal population 42.3, 17.7, and 23.6%, respectively. Endoscopic and pathological findings are generally reported to be consistent in patients with ESRD. The most common abnormality in HD patients was chronic gastritis with a rate of 30% in esophagogastroduodenoscopy and 71.5% in upper gastrointestinal mucosa [21, 38]. Endoscopy was performed in 322 (76.3%) of 422 dialysis patients and gastroduodenal biopsy samples were taken from 260 patients (80.7%). Gastroduodenitis was 49% in endoscopy, gastritis was 52% and duodenitis was 21% on histological examination [39]. In a study where endoscopic findings were chronic gastritis (37%), acute gastritis (20.1%), duodenal ulcer (11.1%), erosive or non-erosive duodenitis (9.3%), gastroduodenitis (5.56%) and GERD (3.7%), histological examination of multiple antral gastric biopsies revealed chronic active gastritis in 51.9% of patients [22]. In another study, gastric mucosal oedema (82.3%), gastritis (23.5%) and increase in number of bi- and multinucleated parietal cells with vacuolation and fragmentation of the cytoplasm (29%), Brunner’s gland hyperplasia (82.4%), duodenitis (70.6%) and gastric metaplasia in duodenum (29.4%) were the main histological changes [40]. The most common pathological finding in our dialysis patients was chronic gastritis (75.5%). The frequencies of intestinal metaplasia and atrophy were 17 and 15.1%, respectively. We did not find dysplasia and neoplasia. Atrophy rate was higher in HD patients than in PD patients, but this difference did not reach statistical significance (22.7% vs. 9.7%). The HP positivity rate in patients with chronic gastritis was 37.5%, while HP was negative in patients without chronic gastritis. In our patients with chronic gastritis, sodium and LDL cholesterol, total protein and albumin levels were higher and urea and ferritin levels were lower.

In our study, the probability of developing chronic gastritis in men was 9.9-fold higher than in women. The presence of gastritis increased the risk of HP positivity by 9.5-fold. Male gender and HP infection are associated with a higher risk of the important endoscopic lesions including esophagitis, gastroduodenal erosions and peptic ulcers, but not age, duration of dialysis, cause of the ESRD, presence of any symptoms, and hemoglobin levels [20]. Interestingly, we found in our study that one unit increase in hemoglobin level increased the risk of duodenitis by 2.21-fold. A hemoglobin > 10.7 g/dL was significantly related to an increased risk of the presence of duodenitis. One unit increase in chloride level reduced the risk of duodenitis by 10%. One-unit increase in ferritin level reduced the risk of HP positivity by 3%. Indeed, HP has been identified as a possible cause of vitamin B12 and iron deficiency in the general population. HP may have an independent role in anemia of HD patients due to gastroduodenal blood loss [41]. HP-positive HD patients may present with lower vitamin B12 blood levels [42].

Gastric and duedonal ulcers were seen in the normal population between 11 and 20% [43]. A study found that HP infection was an independent protective factor for gastric erosion, but it was not associated with other mucosal lesions [26]. In a retrospective study of 827 dialysis patients during the 10-year study period, peptic ulcers were detected in 153 of 481 patients underwent endoscopy. Age, PD, diabetes mellitus, congestive heart failure, low serum albumin and high GGT levels are risk factors for peptic ulcers among ESRD patients [44]. Dialysis patients with peptic ulcer had lower serum albumin levels and higher blood urea nitrogen (BUN) levels than non-ulcer patients. Malnourished patients often show a gradual decrease in BUN levels [31]. Inflammatory stress and malnutrition can impair the gastric mucosa function and induce peptic ulcers. Hypoalbuminemia is one of the strongest predictors for morbidity and mortality in patients with ESRD. Serum albumin concentration correlates not only with lower protein intake, but also with inflammation. Patients with worse gastrointestinal symptoms had lower dietary protein intake. Gastrointestinal symptoms and inflammation were risk factors for lower serum albumin levels [45]. Gastrointestinal symptoms cause reduced food intake in 53% of PD patients and 14% of HD patients. Dietary changes can alleviate symptoms in 34% of PD patients and 9% of HD patients [46]. However, the increased albumin level in our patients increased the risk of chronic gastritis by 8.83-fold. In fact, a good diet that leads to an increase in albumin and urea levels may increase gastric complaints and lesions.

The incidence of HP varies between 24 and 32% in general population [47]. HP positivity was found in 28.3% of our patients. HP positivity rate in PD patients was significantly higher than in HD patients (38.7% vs. 13.6%). The rate of HP infection in uremic patients varies between 24 and 73% in different studies [3, 9, 19, 22, 25, 26, 46, 48]. The prevalence of HP positivity in ESRD patients was significantly lower than those of non-CKD patients (27.5–38.1% vs. 56–67.4%) [26, 34], but it was not confirmed in all studies [2, 25, 48]. Different studies have reported a relationship between the presence of HP with chronic active gastritis, non-erosive esophagitis and gastric metaplasia [19, 22, 41, 49, 50]. Among peptic ulcer disease, HP infection rate in the CKD (58.5%) and ESRD (56.2%) patients was lower than in non-CKD patients (70.3%) [3]. Upper gastrointestinal endoscopic examination has shown the presence of severe HP pangastritis even in some patients without significant complaints [4]. In recent study, 78.6% of patients had non-HP-related gastritis, 38.9% had duodenitis and 32.2% had HP infection. Duodenitis was associated with tacrolimus use [51]. Interestingly, a lower HP gastritis rate of 9.2 to 29% has been reported in gastric biopsies of kidney transplant patients [16, 48, 52]. However, in a small cohort, kidney transplant recipients have had a higher prevalence of HP infection than dialysis and control dyspeptic patients (62% vs. 34.6 and 43.6%, respectively). The rate of active gastritis in transplant (6.9%) and dialysis (3.8%) groups was lower than in control subjects (31.3%) [53]. In the present cohort, the rate of gastritis in HP-positive patients (86.7% vs. 52.6%) and erosive gastritis rate in HP-negative patients (44.7% vs 13.3%) were higher than the other. There was also a significant difference between chronic gastritis (65.8% vs. 100%) and intestinal metaplasia (0% vs. 23.7%) in HP-positive and negative patients, respectively.

The mean duration of dialysis in HP-positive patients is significantly lower than in HP-negative patients. In our study, median dialysis duration in HP-positive patients was shorter than HP-negative patients, but this difference was not statistically significant (22 vs. 30.3 months). The low number of patients in both groups may be the reason for not reaching statistical significance. Chronic gastritis-related gastric acid hyposecretion may decrease the prevalence of HP in long-term dialysis patients [10]. Other studies have also shown that the prevalence of HP infection decreases significantly as the duration of dialysis prolongs, especially within the first 4 years after the start of dialysis [8, 9, 27]. A meta-analysis of dialysis patients found that the HP infection rate was negatively correlated with the duration of dialysis [54]. Recently, two separate meta-analyzes reported a lower estimated prevalence of HP infection, such as 44% in ESRD patients and 48.2% in CKD patients compared to non-CKD patients [17, 55]. Indeed, there is a direct relationship between the duration of dialysis and the incidence of gastric metaplasia [56]. The presence of HP was associated with the occurrence of histological gastritis in both body and antral mucosa, strongly correlated with acute gastritis and acute on chronic gastritis rather than chronic gastritis [39]. If HP positivity was observed in our study, the risk of gastritis increased 5.71-fold. The decrease in HP infection rate with longer dialysis duration can be explained by several mechanisms. In HD patients, high blood urea levels can contribute to a decreased gastric acid secretion and higher gastric pH, and may inhibit the growth of HP in the gastrointestinal tract. In our study, one-unit increase in serum urea level was shown to reduce the risk of chronic gastritis by 5%, but a single BUN measurement can be misleading. However, an urea ≤112 mg/dL was significantly related to an increased risk of the presence of chronic gastritis.

The usages of antibiotics, proton pump inhibitors or H_2_ receptor antagonists in dialysis patients may inhibit HP growth. Severe gastric mucosal atrophy leading to decreased H^+^ secretion may occur in HD patients secreting inflammatory cytokines. In addition, secretion of inflammatory cytokines in HD patients cause severe chronic gastritis leading to decreased secretion of H^+^, and this affect the colonization of HP in the gastric mucosa [54, 57]. In contrast, some studies have shown that high urea concentrations make the gastric mucosa more susceptible to HP, and uremic patients have a higher infection rate. The high incidence of peptic ulcers in CKD patients is caused by various reasons other than HP infection [58]. Among ESRD patients without symptom, serum pepsinogen I/II ratio was significantly higher in HP-negative patients than in HP-positive patients [26].

In our study, total cholesterol level in HP-positive patients was higher than HP-negative patients. Hypocholesterolemia (< 140 mg/dL) was associated with higher mortality in hemodialysis patients [31]. One-unit increase in total cholesterol level increased risk of HP positivity by 1.04-fold. A total cholesterol > 243 mg/dL was significantly related to an increased risk of the presence of HP positivity. Statins reduce cellular cholesterol and risk of HP infection by decreasing HP burden in macrophages, and consequently alleviate inflammation caused by HP [58]. Also, depletion of cholesterol has been demonstrated to attenuate CagA-induced pathogenesis [59]. Adding statin to the standard triple therapy may improve the eradication rate of HP infection [60].

Many studies did not find a significant relationship between anemia and iron deficiency with HP positivity and other endoscopic findings except bleeding [61]. We detected significant relationship between duedonitis and lower hemoglobin. We evaluated anemia as the primary outcome of CKD, independent of HP positivity.

Chronic gastritis described in approximately 50% of uremic patients [62]. We found it significantly too. Inflammation and medications may affected this condition. Hypoalbuminemia detected in 81% of patients with gastropathy [63]. Hypoalbuminemia becomes evident when kidney failure progresses, depending on nutrition and inflammatry process [45]. In our study, We found albumin levels low, as in the literature.

## Conclusions

Gastrointestinal non-ulcerous lesions are common in dyspeptic dialysis patients. Dialysis modality may affect the frequency of some lesions. Although our study included relatively low number of patients in a single center, HP positivity was more common in PD patients than in HD patients. Chronic gastritis was observed in most patients (75.5%) and 37.5% of them were HP-positive. In our study, we only highlighted patological data and findings. Gender was only significantly in patients with chronic gastritis. The results of our study also show that it is beneficial in HP eradication to control serum total cholesterol levels in patients with HP positivity. Even if they have dyspeptic symptoms, it may be useful to evaluate dialysis patients by endoscopic examination and to treat those with gastrointestinal lesions.

The number of patients was one of our most important limitations. We do not apply upper gastrointestinal system endoscopy routinely in our institution, except patients who have active complaints and symptoms.

## Data Availability

All or part of the data in this study did not publish and present in another journal or meeting. The datasets used and/or analysed during the current study are available from the corresponding author on reasonable request.

## Abbreviations

PD: Peritoneal dialysis
HD: Hemodialysis
HP: *Helicobacter pylori*
CKD: Chronic kidney disease
MALT: Mucosa Associated Lymphoid Tissue
ROC: Receiver operator characteristic
HIV: Human immunodeficiency virus
HCV: Hepatitis C
SD: Standart deviation
Min: Minimum
Max: Maximum
CRP: C reactive protein
LDL: Low dansity lipoprotein
TSAT: Transferrin saturation
HbA1C: Hemoglobin A1C
GGT: Gamma glutamyltransferase
ESRD: End stage renal disease
GERD: Gastroesophageal reflux disease
BUN: Blood urea nitrogen
